# Item reduction and validation of the Chinese version of diabetes quality-of-life measure (DQOL)

**DOI:** 10.1186/s12955-018-0905-z

**Published:** 2018-04-27

**Authors:** Xuejing Jin, Gordon G. Liu, Hertzel C. Gerstein, Mitchell A. H. Levine, Kathleen Steeves, Haijing Guan, Hongchao Li, Feng Xie

**Affiliations:** 1 Department of Health Research Methods, Evidence, and Impact, McMaster University, 1280 Main St W, Hamilton, ON L8S 4K1 Canada; 2 China Center for Health Economic Research, Peking University, Beijing, 100800 China; 3 National School of Development, Peking University, Beijing, 100800 China; 4 Department of Medicine, McMaster University, Hamilton, ON L8S 4K1 Canada; 5 Department of Sociology, McMaster University, Hamilton, ON L8S 4K1 Canada; 6 School of Pharmaceutical Science, Peking University, Beijing, 100800 China; 7 School of International Pharmaceutical Business, China Pharmaceutical University, Nanjing, 211198 Jiangsu China; 8 Centre for Evaluation of Medicines, Father Sean O’Sullivan Research Centre, St. Joseph’s Healthcare Hamilton, Hamilton, L8N 4A6 Canada

**Keywords:** Item response theory, Classical test theory, Factor analysis, Diabetes, Quality of life, Psychometrics

## Abstract

**Background:**

The Diabetes Quality-of-Life (DQOL) Measure is a 46-item diabetes-specific quality of life instrument. The original English version of the DQOL has been translated into Chinese after cultural adaption, and the Chinese DQOL has been validated in the Chinese diabetic patient population and used in diabetes-related studies. There are two recognized problems with the Chinese DQOL: 1) the instrument is too long, and 2) the non-response rate of certain items is relatively high. This study aimed to develop and validate a short version for the Chinese DQOL.

**Methods:**

Item reduction was conducted based on the classical test theory (CTT) and item response theory (IRT), each combined with exploratory factor analysis (EFA). The confirmatory factor analysis (CFA) and Spearman correlation coefficient were employed in validating the short versions.

**Results:**

Both the study sample (*n* = 2,886) and the validation sample (*n* = 2,286) were from a longitudinal observation study of Chinese type 2 diabetic patients. The CTT kept 32 items, and the IRT kept 24 items from the original 46-item version. The two short versions were comparable in psychometric properties.

**Conclusion:**

The 24-item IRT-based short version of the Chinese DQOL was selected as the preferred short version because it imposes a lower burden on patients without compromising the psychometric properties of the instrument.

**Electronic supplementary material:**

The online version of this article (10.1186/s12955-018-0905-z) contains supplementary material, which is available to authorized users.

## Background

The global prevalence of diabetes mellitus (DM) in adults was 9.1% (415 million people) in 2015, which makes DM one of the most common chronic diseases around the world [1]. Diabetes-related complications, receiving blood glucose control therapies, and dealing with hypoglycemic agents and/or insulin adverse reactions seriously affect patients’ (and their family members’) health-related quality of life (HRQoL) in both physical and psychological ways [2, 3]. Hence, diabetic patients’ HRQoL outcomes have been increasingly recognized as valuable and essential information to obtain in the fields of clinical research and diabetes management.

Diabetic patients’ HRQoL are measured by generic or diabetes-specific instruments [4]. Diabetes-specific instruments, as designed to focus on diabetes specific conditions, are more sensitive to diabetes-symptoms-related impacts on life and quality of life than generic instruments [5]. The Diabetes Quality-of-Life Measure (DQOL) is one of the most commonly used diabetes-specific instruments [6, 7]. It was developed and validated to compare two treatment regimens for chronic complications in patients with diabetes in the Diabetes Control and Complications Trial (DCCT) [8, 9]. The DQOL contains a total of 46 items, and all the items are categorized into one of the following four domains: life satisfaction (15 items), diabetes impact (20 items), social/vocational related worries (7 items), and diabetes related worries (4 items). The DQOL adopts a 5-point Likert scale for its response options. The scores range from 1, labeled as “very satisfied,” to 5, labeled as “very dissatisfied,” for items in the life satisfaction domain; from 1, labeled as “never impacted,” to 5, labeled as “always impacted,” for items in the diabetes impact domain; and from 1, labeled as “never worried,” to 5, labeled as “always worried,” for the social/vocational related and diabetes related worries domains.

The DQOL has been translated into five languages, including Chinese [10]. This measure was first translated and adapted for Chinese-Canadians who lived in the Toronto area by Cheng et al. [11, 12]. They removed 10 privacy-related (e.g. sexual life) items from the original DQOL and added six items regarding diet, worrying about death and so on. However, there was not sufficient psychometric evidence to support the cultural adaptation in Cheng et al.’s study [11], and the translation and validation were conducted based on an immigrant population, which cannot necessarily be generalized to the entire Chinese diabetic patient population. Ding et al. translated and adapted the DQOL for the Chinese population based on a sample of Chinese patients with diabetes who lived in Mainland China [13], and conducted validation of the Chinses DQOL on a separate sample of Chinese patients with type 2 DM living in Mainland China [14]. The wording of seven items was changed in Ding et al’s adaptation (see Additional file 1). Currently, the Chinese DQOL translated and adapted by Ding et al. has been used in diabetes-related clinical studies in China [15–17]. During its application among the Chinese diabetic patient population, the Chinese DQOL has exposed some of its own issues [18]. First, the non-response rate of certain privacy-related items was relatively high; and second, interviewees complained that the instrument was too long [19, 20]. In order to solve these issues, developing and validating a short version of the Chinese DQOL is necessary.

The classical test theory (CTT) and the item response theory (IRT) are two commonly used psychometric theories in conducting item selection and reduction for measures; however, these two theories work based on different assumptions and statistical approaches, and both have shortcomings [21, 22]. More specifically, the CTT assumes that each respondent has a true total score, T (latent variable), and each item is a representative of the score T; while the IRT follows the assumptions that the latent trait of a measure is unidimensional and all items are conditionally independent of each other. Generally, CTT tests the difficulty and discrimination at the item level and the reliability at the whole measure level; while IRT uses a set of logistic regression models to estimate the “discrimination,” “location,” and “information” for each item [21, 22]. The CTT is limited by the sample and item/test dependence and equal error (of measurement across examinees) assumption [21, 22]. The IRT overcomes these shortcomings but requires for large sample sizes for model fitting [21, 22]. There is no generally accepted approach or standard for item reduction. Currently, researchers have been using the IRT alone [23], the combination of the IRT and factor analyses [24, 25], or the combination of the CTT and factor analysis [26, 27] when selecting or reducing items.

Therefore, the present study aims to use both the CTT and IRT combined with factor analysis to derive and validate a short version of the Chinese DQOL, which can be rapidly administered in practice and can reduce response burden on patients.

## Methods

### Sample and data

We used the data from a Chinese community-based longitudinal survey of clinically diagnosed type 2 diabetic patients (T2DP) from five cities: Beijing, Chengdu, Guangzhou, Nanjing, and Shenyang. Patients were recruited and interviewed between December 2010 and October 2011, and followed every three months over a one-year study period. The Chinese DQOL and the EQ-5D-3L were administered at the baseline and at 12-months. Demographic, social-economic and diabetic-related information was also collected. We used the baseline data as the study sample for item reduction analysis, and the one-year end follow-up data as the validation sample to test the short versions of the Chinese DQOL reduced by CTT and IRT.

### Reduction based on the classical test theory

Three steps were used to reduce the number of items based on the CTT. The first step tested each item at the individual item level, and the second and third steps examined the items at the whole measure or domain level. The following provides the details of the tests in each step and the corresponding item removal criteria.

#### Step 1. Item level tests

We tested three item level properties for each of the 46 items in this step, i.e., missing rate, item score mean, and item score standard deviation (SD).

Items which are unclear, ambiguous, or potentially embarrassing usually have a higher chance to have high non-response rate issues. This kind of item can provide very limited useful information, and its results are hard to interpret [21]. The exclusion criterion for the missing rate was higher than 5% [28].

In the CTT, item difficulty and discrimination are often evaluated in item level testing; however, most of the item difficulty and discrimination indexes are designed to test dichotomous items and can hardly be applied to test Likert items [29]. Norman has provided compelling evidence on the appropriateness of using descriptive statistics and parametric methods to test Likert items [30, 31]. The mean and SD of an item can provide fundamental information on whether the item can provide useful information or not [32]. For example, if the mean score is 4.7 for a 5-point Likert item (score range: 1 to 5), then the item is left-skewed and may not be able to provide the information it was designed to collect. In addition, if the SD of an item is low, then the item has low variability and it may not be useful either. There are no generally accepted criteria for the item level test using mean and standard deviation, and we used the most lenient criteria reported in the existing studies. We used the lowest score option plus 20% of the score range and the highest score option minus 20% of the score range to define the cut point of the exclusion criterion in terms of item score mean [21, 33, 34]. The lowest and highest score options for each item is 1 and 5, respectively, and the score range for each item is 4. Thus, the exclusion criterion for the item score mean was lower than 1.8 or higher than 4.2. The exclusion criterion for the item score SD was smaller than one-sixth of the score range, i.e., 0.67 (1/6*4) [21, 33–35].

Any item that met any two or more of the three exclusion criteria was removed from the measure. In addition, any item with a missing rate higher than 10% was removed regardless of the results of the other two criteria.

#### Step 2. Exploratory factor analysis

In this step, exploratory factor analysis (EFA) was employed on the remaining items to examine the underlying structure of the measure and remove items with low factor loadings on common factors.

More specifically, Bartlett’s test of sphericity [36] and Kaiser-Meyer-Olkin’s (KMO) measure of sampling adequacy [37] were conducted before conducting the EFA. Since the training sample violated the assumption of multivariate normality, we employed the principal-factor extraction method [38]. A scree plot was used to identify the number of factors [39]. Oblique rotation method was used in the EFA since the DQOL items were not completely unrelated to each other [40]. In this step, any item with a factor loading less than 0.3 was removed [41].

#### Step 3. Internal consistency reliability

Internal consistency reliability was tested in terms of the corrected item-total correlation and Cronbach’s alpha [29]. Both tests were conducted at the factor level based on the results of the EFA in step 2.

Since there is no standard scoring method for the Chinese DQOL, we used the patients’ mean score of the items in each factor as the “factor score” when calculating the corrected item-total correlation. For each item, the corrected item-total correlation was calculated as the Pearson correlation coefficient between the item score and the mean score of the rest of the items in the factor this item belonged to. A larger corrected item-total correlation coefficient indicates better internal consistency reliability. The exclusion criterion was a correlation coefficient smaller than 0.3 [42]. For the Cronbach’s alpha, the exclusion criterion was that the Cronbach’s alpha of the factor increased after removing an item [43].

In this step, any item that met one or more of these two exclusion criteria was removed from the measure. An additional EFA was used to check if the factor structure changed after this step; if so, the new factor structure would be used as the final structure of the short version developed based on the CTT.

### Reduction based on the item response theory

One of the basic assumptions of the IRT is unidimensionality [44]; however, DQOL was designed to measure multiple aspects of burden that diabetes places on patients. In order to conduct the IRT analysis without violating the assumption of unidimensionality, we employed EFA in the first place to re-identify the potential dimensional structure of the original Chinese DQOL and then fitted the sets of IRT models for each individual dimension. Details of the two steps are as follows.

#### Step 1. Exploratory factor analysis

Similar to the EFA analysis process adopted under the CTT reduction approach, Bartlett’s test and KMO test were carried out for testing the sphericity and sampling adequacy, respectively, before implementing the EFA under the IRT reduction approach. Number of factors was identified by a scree plot generated based on the 46 Chinese DQOL items. Then principal-factor extraction method and oblique rotation method were employed to conduct the EFA. In this step, any item with a factor loading of less than 0.3 was removed.

#### Step 2. Item response theory analysis

The graded response model (GRM), which is a type of item response model for items with ordered response options [45], was employed in this step to analyze the remaining items within each factor identified in step 1. The GRM was first introduced by Samejima [45]. It models each item with its own discrimination parameter and a set of parameters that identify the boundaries between the ordered options using a logistic regression approach. The item information functions (IIFs) were built based on the fitted GRMs to evaluate the “information”, i.e., reliability, each item contributed to the factor. A larger amount of information an item can provide indicates a better item it is. The GRM and IIF formulas are presented in the Appendix.

In this step, any item that had an estimation of discrimination parameter less than 1.0 [46] and provided item information less than 0.5 was removed from the measure [25]. An additional EFA was also conducted to check the factor structure; and if the structure changed after this step, the new factor structure would be used as the final structure of the short version developed based on the IRT.

### Validating and comparing the two short versions of the Chinese DQOL

We evaluated and compared the two short versions at three aspects, i.e., performance in the confirmatory analysis (CFA), correlation with EQ-5D, and the magnitude of reduced response burden.

#### Confirmatory factor analysis

The CFA was employed to validate the structure of the two short versions of the Chinese DQOL. We specified that the domains were correlated with each other and employed maximum likelihood estimation in the CFA. Two statistics produced by the CFA were used to compare the performance of the two versions: standardized root mean squared residual (SRMR) and comparative fit index (CFI).

The SRMR is the square root of the difference between the residuals of the sample covariance matrix and the proposed covariance model. It ranges from 0 to 1, and a smaller value indicates a better fit [47]. The CFI compares the sample covariance matrix with this null model based on the assumption that all latent variables (factors) are uncorrelated. The CFI ranges from 0 to 1, and a larger value indicates a better fit [47]. Since the variation of performance among fit indices, according to Hu and Bentler’s two-index presentation strategy [48], we adopted the SRMR as the fundamental fit index and the CFI and as a supplementary index.

#### Correlation with the EQ-5D

We tested the construct validity of the two reduced versions of the Chinese DQOL against the EQ-5D-3L index and EQ visual analogue scale (EQ-VAS).

The EQ-5D-3L is a widely used preference-based generic quality of life instrument which has 5 questions that ask about whether there are any problems in: mobility, self-care, usual activities, pain/discomfort and anxiety/depression. Each question has three response levels, i.e., no problems, some (or moderate) problems, and extreme problems (or unable to). Patients’ EQ-5D-3L responses were converted in to EQ-5D-3L values by using the Chinese EQ-5D-3L value set [49]. The EQ-VAS records the patient’s self-rated health on a vertical, visual analogue scale which ranges from 0 (the worst imaginable health state) to 100 (the best imaginable health state) [50].

Spearman’s correlation coefficients between the EQ-5D-3L index and the mean score of each one of the two short versions of the Chinese DQOL were calculated respectively. The correlation coefficients between the EQ-VAS and the two short versions were also calculated individually. A larger correlation coefficient indicates a higher construct validity [28, 29].

#### Final short version selection

The short version which performed better in both the CFA and had higher correlation with EQ-5D was selected as the final short version of the Chinese DQOL. In the event of any conflict between the CFA and the correlation analysis results, we selected the short version reduced more response burden as the final short version of the Chinese DQOL.

All statistical analyses were conducted with a two-tailed test at the significance level of 0.05 in STATA 14.2 (StataCorp LP, Texas, USA).

## Results

### Sample

A total of 2886 patients were recruited and interviewed at the baseline. The mean age and diabetes duration of the study sample was 61.15 years and 7.94 years, respectively. Among all patients, 55.68% were female, 64.10% were retired, and 16.18% had used insulin in the last 6 months. The mean scores of the EQ-5D-3L index, VAS, and the Chinese DQOL (mean score of the 46 items) were 0.89, 72.71, and 2.07, respectively (Table 1). In the validation analyses, the CFA and the calculation of the EQ-5D-3L index only employed observations without missing data. Because of this, our validation sample only included patients with no missing values on responses to the 5 questions of the EQ-5D and to the DQOL items kept after the item reduction based on the CTT and IRT. Of the 2542 patients who completed the year-end follow-up, 2286 were included in the validation sample (Table 1). Compared to the study sample, the validation sample had a higher proportion of people who were older, retired, and used insulin (Table 1).

**Table 1:** Patients’ baseline demographic and diabetes-related information

	Study sample (*N* = 2886)	Validation sample (*N* = 2286^a^)	*P*-value^b^
Age mean (SD), years	61.15 (11.42)	61.84 (11.25)	0.030
Female	1607 (55.68)	1269 (55.51)	0.910
Diabetes duration mean (SD), years	7.94 (6.75)	7.69 (6.33)	0.174
Retired	1850 (64.10)	1544 (67.54)	0.010
Used insulin in the last 6 months	467 (16.18)	476 (20.82)	< 0.001
Controlled diet	2580 (89.40)	2036 (89.06)	0.718
EQ-5D-3 L index mean (SD)	0.89 (0.14)	0.89 (0.13)	0.999
EQ-ED VAS mean (SD)	72.71 (15.46)	73.00 (15.48)	0.503
DQOL score mean (SD)^c^	2.07 (0.38)	2.07 (0.39)	0.999

The values presented are numbers (percentage) unless otherwise stated

^a^A total of 2542 patients finished the last round follow-up at the year end. Only observations with no missing data on the EQ-5D-3L and DQOL questions were included in the validation sample

^b^T-test for mean and Chi-square test for frequency

^c^The DQOL score was calculated from the mean of the 46 items

*SD* Standard deviation; *VAS* Visual analogue scale; *DQOL* Diabetes quality-of-life measure

### Item reduction results

Tables 2 and 3 show the item reduction results based on the CTT and IRT, respectively. A total of 14 and a total of 22 items (details see supplementary materials) were removed from the Chinese DQOL based on the CTT and IRT, respectively.

**Table 2:** Item reduction results based on the CTT

Item No.	Step 1. Item level tests			Step 2. EFA		Step 3. Internal consistency reliability	
	Missing rate (%)	Mean score	SD	Factor 1	Factor 2	Item-total corrected correlation coefficient^b^	Cronbach’s alpha^b^
	Missing rate > 5%^a^	< 1.8 or > 4.2^a^	< 0.67^a^	Factor loading < 0.3^a^		*r* < 0.3^a^	Increased after remove^a^ (Factor 1: 0.884 Factor 2: 0.822)
1	0.312	2.081	0.711	0.384	0.528^c^	0.667	0.764
2	0.277	2.132	0.693	0.337	0.519^c^	0.648	0.772
3	0.243	2.078	***0.649***	0.357	0.550^c^	0.695	0.751
4	0.104	2.174	0.725	0.388	0.448^c^	0.564	0.811
5	0.104	2.653	0.926	0.394^c^	0.172	0.348	0.882
6	0.069	3.129	0.994	0.518^c^	0.015	0.492	0.879
7	0.069	2.563	0.811	***0.297***	***0.170***	Removed	
8	0.035	2.740	1.090	0.398^c^	0.133	0.367	0.882
9	0.139	2.112	0.679	0.420^c^	0.262	0.360	0.882
10	***14.969***	2.449	0.769	Removed			
11	0.485	2.161	0.692	0.485^c^	0.234	0.419	0.880
12	0.035	2.594	0.912	0.406^c^	0.106	0.361	0.882
13	0.312	2.419	0.900	0.398^c^	0.171	0.345	0.882
14	0.277	2.290	0.748	0.492^c^	0.236	0.434	0.880
15	0.035	2.158	***0.656***	0.566^c^	0.266	0.498	0.879
16	0.139	2.466	1.201	0.557^c^	−0.137	0.539	0.878
17	0.069	***1.727***	0.953	0.431^c^	− 0.166	0.415	0.881
18	0.035	2.321	1.072	0.381^c^	−0.068	0.379	0.882
19	0.035	2.556	1.075	0.534^c^	−0.018	0.520	0.879
20	0.069	2.580	1.223	0.608^c^	−0.165	0.597	0.877
21	0.035	2.631	1.225	0.423^c^	0.060	0.396	0.882
22	0.069	***1.724***	0.892	0.492^c^	−0.124	0.468	0.880
23	0.035	2.729	1.135	***0.230***	***0.006***	Removed	
24	0.035	2.960	1.239	0.381^c^	−0.132	0.381	0.882
25	***14.414***	1.875	0.968	Removed			
26	1.802	***1.581***	0.961	***0.245***	***−0.173***	Removed	
27	0.416	***1.790***	1.012	0.487^c^	−0.144	0.451	0.880
28	0.104	1.843	1.047	0.514^c^	−0.152	0.490	0.879
29	0.035	2.445	1.176	***0.185***	***−0.228***	Removed	
30	0.208	1.882	0.977	0.574^c^	−0.192	0.526	0.878
31	0.035	2.343	1.079	***0.222***	***−0.187***	Removed	
32	0.069	***1.297***	***0.594***	Removed			
33	0.104	2.499	1.260	0.355^c^	−0.089	0.342	0.883
34	0.035	***1.781***	0.945	***0.234***	***−0.029***	Removed	
35	***8.073***	***1.136***	***0.415***	Removed			
36	2.495	***1.229***	***0.593***	Removed			
37	0.728	1.888	1.147	0.429^c^	−0.166	0.420	0.881
38	3.222	***1.262***	0.685	0.314^c^	−0.177	***0.294***	0.883
39	2.668	***1.311***	0.762	0.332^c^	−0.197	0.305	0.883
40	4.089	***1.134***	***0.431***	Removed			
41	3.915	***1.213***	***0.633***	Removed			
42	0.312	***1.641***	1.018	0.424^c^	−0.263	0.414	0.881
43	0.104	1.998	1.151	0.519^c^	−0.264	0.508	0.879
44	0.069	***1.551***	0.908	0.441^c^	−0.225	0.425	0.881
45	0.104	2.632	1.317	0.502^c^	−0.182	0.487	0.880
46	0.104	***1.353***	0.720	0.427^c^	−0.250	0.414	0.881

Bold and italic number indicates the item failed the corresponding test

Dashed box indicates the item(s) was removed from the scale

^a^Exclusion criteria

^b^Total scores were calculated as the corrected mean score of the factor

^c^indicates which factor the item belongs to based on the EFA

*CTT* Classical test theory, *EFA* Exploratory factor analysis, *SD* Standard deviation, *N/A* Not applicable

**Table 3:** Item reduction results based on the IRT

Item No.	Step 1. EFA		Step 2. IRT	
	Factor 1	Factor 2	Discrimination	Item information function^b^
	Factor loading< 0.3^a^		< 1^a^	< 0.5^a^
1	0.356	0.421^c^	2.830	> 2, < 2.5
2	0.313	0.405^c^	2.848	> 2, < 2.5
3	0.342	0.407^c^	3.496	> 3, < 3.5
4	0.357	0.368^c^	1.987	> 1, < 1.5
5	0.361^c^	0.248	***0.778***	***< 0.2***
6	0.506^c^	0.115	1.097	< 0.4
7	***0.274***	***0.181***	Removed	
8	0.369^c^	0.258	***0.769***	***< 0.2***
9	0.427^c^	0.203	1.024	< 0.3
10	0.343^c^	0.153	***0.768***	***< 0.2***
11	0.470^c^	0.160	1.211	< 0.5
12	0.365^c^	0.184	***0.854***	***< 0.3***
13	0.373^c^	0.171	***0.884***	***< 0.3***
14	0.466^c^	0.218	1.147	< 0.4
15	0.544^c^	0.207	1.485	> 0.6, < 0.7
16	0.544^c^	0.045	1.328	> 0.5, < 0.6
17	0.456^c^	−0.128	1.237	< 0.5
18	0.368^c^	0.077	***0.801***	***< 0.2***
19	0.494^c^	0.186	1.178	< 0.5
20	0.592^c^	0.031	1.567	< 0.5
21	0.394^c^	0.205	***0.850***	***< 0.3***
22	0.491^c^	−0.063	1.506	> 0.6, < 0.7
23	***0.220***	***−0.023***	Removed	
24	0.352^c^	0.068	***0.793***	***< 0.2***
25	0.404^c^	−0.076	***0.991***	***< 0.3***
26	***0.262***	***−0.150***	Removed	
27	0.485^c^	−0.052	1.429	> 0.6, < 0.7
28	0.519^c^	−0.041	1.517	> 0.7, < 0.8
29	***0.173***	***−0.059***	Removed	
30	0.575^c^	−0.087	1.666	> 0.8, < 0.9
31	***0.212***	***−0.017***	Removed	
32	0.346^c^	−0.242	***0.998***	***< 0.3***
33	0.345^c^	0.042	***0.762***	***< 0.2***
34	***0.271***	***−0.060***	Removed	
35	***0.214***	***−0.197***	Removed	
36	0.383^c^	−0.351	1.068	< 0.4
37	0.449^c^	−0.162	1.099	< 0.4
38	0.410	−0.514^c^	***0.321***	***around 0***
39	0.403	−0.441^c^	***0.303***	***around 0***
40	0.330	−0.461^c^	***0.360***	***around 0***
41	0.372	−0.510^c^	***0.289***	***around 0***
42	0.433^c^	−0.182	1.224	< 0.5
43	0.526^c^	−0.089	1.357	> 0.5, < 0.6
44	0.450^c^	−0.180	1.306	> 0.5, < 0.6
45	0.479^c^	0.021	1.048	< 0.4
46	0.465^c^	−0.280	1.515	> 0.7, < 0.8

Bold and italic number indicates the item failed the corresponding test

Dashed box indicates the item(s) was removed from the scale

^a^Exclusion criteria;

^b^Highest point on the item information function curve;

^c^indicates which factor the item belongs to base on the EFA

*IRT* Item response theory, *EFA* Exploratory factor analysis

In step 1 of the reduction based on the CTT, two items, item #10 (satisfied with sex life) and item #25 (interferes with sex life) were removed from the measure because their missing rates were higher than 10%. Item #32 (being teased because of having diabetes), item #36 (worry about marriage), item #40 (worry about completing education), and item #41 (worry about unemployment) were removed because of their low mean scores (all < 1.8) and small SDs (all < 0.67). Item #35 (hide having an insulin reaction) was removed because of the high missing rate (8.07%) and low mean score and small SD. In step 2, the EFA identified two factors among the remaining items. Item #7 (satisfied with knowledge about diabetes), item #23 (feel good about yourself), item #26 (interfere with riding a bike or using a machine), item #29 (explain what it means to have diabetes), item #31(tell others about your diabetes), and item #34 (eat something you shouldn’t rather than tell someone that you have diabetes) were removed due to low factor loadings (< 0.3). In step 3, item #38 (worry about whether you can get a job you want) was removed because of the low correlation with the mean score of the factor it belonged to. The factor structure identified in Step 2 remained the same after removing item #38 in Step 3.

In the reduction based on the IRT, the EFA identified 2 factors of the 46 DQOL items, and removed items #7, #23, #26, #29, #31, and #34 because their factor loading were all smaller than 0.3. In step 2, item #5 (satisfied with the flexibility of the diet), item #8 (satisfied with sleep), item #10, item #12 (satisfied with the appearance of your body), item #13 (satisfied with the time spent on exercising), item #18 (low blood sugar reactions), item #21 (bad night’s sleep), item #24 (feel restricted by diet), item #25, item #32, item #33 (feel that because of diabetes you go to the bathroom more than others), item #38, item #39 (worry about the pension), item #40, and item #41 were removed in the IRT analysis due to their item discrimination being smaller than 1 and their item information being lower than 0.5 (Table 3). The factor structure identified in the EFA remained the same after the IRT analysis.

### Validation results

Table 4 shows the validation results of the two short versions of the Chinese DQOL. In the CFA, the two short versions had similar SRMRs (0.078, after rounding, for both short versions) which were also similar to that of the original Chinese DQOL (SRMR = 0.077). The short version based on the IRT had a larger CFI (0.726) than that of the version reduced based on the CTT (CFI = 0.630). The CFI of each short versions was larger than that of the original Chinese DQOL (CFI = 0.616).

**Table 4:** Validation result^a^

	Confirmatory factor analysis		Spearman correlation coefficient	
	SRMR	CFI	ρ(EQ-5D-3L)	ρ(EQ-VAS)
Short version based on the CTT (32 items)	0.078	0.630	−0.298	−0.288
Domain 1			−0.260	−0.260
Domain 2			−0.069	−0.148
Short version based on the IRT (24 items)	0.078	0.726	−0.288	−0.269
Domain 1			−0.240	−0.242
Domain 2			−0.069	−0.148
Original Chinese DQOL (46 items)	0.077	0.616	−0.276	−0.273

^a^Calculations based on a total of 1350 observations without missing values on all the five EQ-5D-3 L questions and all the 46 DQOL items

*CTT* Classical test theory, *IRT* Item response theory, *DQOL* Diabetes quality-of-life measure, *SRMR* Standardized root mean squared residual, *CFI* Comparative fit index, *ρ* Spearman’s correlation coefficient, *VAS* Visual analogue scale

The absolute Spearman’s correlation coefficient between the CTT reduced version of the DQOL and the EQ-5D-3L index scores was 0.298, which was higher than that (ρ = 0.288) between the IRT reduced version and the EQ-5D-3L index scores. Both reduced versions had a higher correlation with the EQ-5D-3L index scores than the original Chinese DQOL (ρ = 0.276). In terms of testing using the EQ-VAS, the CTT-based short version had a higher correlation (ρ = 0.288) than the original version (ρ = 0.273), and the IRT-based short version had a slightly lower correlation (ρ = 0.269) than the original version.

## Discussion

This study shortened the 46-item Chinese version of the DQOL based on two psychometric theories, the CTT and IRT, each combined with the EFA, respectively. The two short versions were validated using the CFA and Spearman correlation coefficients. The CTT provided a short version of the Chinese DQOL with 32 items kept, and the IRT provided a short version with 24 items kept. Among the 14 items removed based on the CTT, 13 were removed based on the IRT as well.

There are few published studies we can compare our results with. Two items related to sexual life had high missing rates in our study, and were removed from the measure in the reduction processes based on both the CTT and IRT. This was consistent with the translation and cultural adaptation study conducted in 1999 among Chinese diabetic patients lived in Canada [12]. The high missing rate of the sexual life items is still in line with the findings in translation and cultural adaptation studies published after 2015 in other disease specific measures among the Chinese population [51]. Chinese people, especially those who are middle-aged and elderly, tend to be hesitant to talk about sex-related topics because of their relatively conservative culture background [52].

Three working and education-related items, i.e., items #38, #40, and #41, had low mean scores (Table 2) and low discriminations (Table 3), and were removed based on both the CTT and IRT. This was because most patients (64.10%) in our training sample were retired, and were not worried about working and education-related issues. These items were also removed according to the expert advice in Cheng’s [11, 12] translation and cultural adaptation study.

The insulin reaction item (item #35) was removed based on both the CTT and IRT. This was because the majority of the patients in the study sample had not used insulin in the last 6 months. Similarly, the diet-related item (item #34) was also removed mainly because the majority of the patients in the study sample controlled their diet by eating healthy food and balancing their amount of food intake due to their diabetes.

In Ding’s [13] translation and cultural adaptation analysis, the descriptive of item 26, “How often does your diabetes keep you from driving a car or using a machine (e.g., a typewriter)?” was changed into “How often does your diabetes keep you from riding a bike or being a typist?” This item was removed because of low factor loading in both reduction processes. Ding et al. changed the “driving a car” into “riding a bike” because civilian vehicle ownership in China was relatively low in the 1990’s, and bicycles were the main means of transportation for ordinary people. However, civilian vehicle ownership in 2012 increased by 544% from 1999 [53], which may make this change in descriptive out-of-date. In addition, typewriters have long been replaced by laptops and other smart electronics which are indispensable in contemporary Chinese people’s daily lives. Therefore, further studies examining the performance of a more up-to-date descriptive, for example, “How often does your diabetes keep you from driving a vehicle or using a computer or smart phone?” are necessary.

There were 9 items that were removed in the IRT-based short version but kept in the CTT-based short version. All of these items were removed due to their low estimated discrimination and item information in the IRT analysis. One of the possible reasons for this difference is that the reduction results were impacted by the exclusion criteria we employed. Even though we used the most lenient fail criteria reported in existing studies for each, respectively, the item reduction results may still not be comparable due to the different statistical approaches applied in the two different theories.

Items #1 to #4 (satisfaction level of “the amount of time it takes to manage your diabetes,” “the amount of time you spend getting a checkup,” “the time it takes to determine your sugar level,” and “your current treatment”) were the only four treatment and diabetes management related items in the DQOL. These items loaded onto the same factor in our EFA. The rest of the 28 items in the CTT-based short version and the rest of the 20 items in the IRT-based short version belonged to the other factor, respectively. This was different than the original Chinese DQOL which has four domains. The CFA and correlation soefficients showed that the structures of the two short versions were comparable to the original version. In addition, we did not emphasize the name of the factors identified in the short versions since the present study aimed to focus on reducing the number of items for the Chinese DQOL. Content and face validity of the short versions should be examined in further studies to optimize the structure and rename the factors of the short versions.

The often-used fit indexes in the CFA are the Chi-square test and the root mean square error of approximation (RMSEA) [47]. In the present study, we employed the SRMR and CFI instead of the Chi-square test and RMSEA. The Chi-square test result is affected by the number of parameters, complexity of the model, and the sample size [54]. Adding more parameters into the model can improve the RMSEA as well [55]. Our two short versions of the Chinese DQOL had different numbers of items; therefore, the Chi-square test and RMSEA were inappropriate to use for comparing the CFA results of these two short versions. The SRMR is not affected by the model complexity and the number of parameters. The CFI is affected by the number of parameters added into a model, but is relatively more stable than the Chi-square test and RMSEA.

Because the two short versions of the Chinese DQOL were comparable in the validation analysis, and we did not have a hierarchy in these two criteria, we selected the short version based on the IRT (24 items) as a preferred short version for two other reasons. First, this shorter version imposes a lower burden on patients without compromising its measurement properties [56]. Second, theoretically, as a modeling statistic approach, the parameters estimated from a set of IRFs can be generalized to the entire population the study sample comes from; however, as a person statistic approach, all CTT test results can only be specified to the given study sample [57].

There are some limitations in our study. First, the training and validation samples were not independent. We did not have a truly external validation sample for our study. Second, our training sample only contained community-based patients, and most of them did not use insulin. This sample was relatively healthier than the diabetic population who had more comorbidities, was inpatient, or using insulin; therefore, our results cannot necessarily be generalized to the entire diabetic patient population. At the validation stage of this study, the CFI value of both versions did not meet the generally accepted criteria for good fit, i.e., CFI > 0.90 [47]. Even though the CFI was used as a supplementary index to evaluate the model fit, this result still added uncertainty to our conclusions. Other psychometric properties such as test-retest reliability of the short version of the Chinese DQOL need to be examined in future studies.

## Conclusions

The version developed based on the IRT retained 24 items was selected as our preferred short version of the 46-item Chinese DQOL. It can impose a lower response burden on patients in practice without compromising the psychometric properties. Further research validating the IRT-based short version of Chinese DQOL is needed.

### Additional file

Additional file 1: Original Chinese DQOL and short versions based on the CTT and IRT. (DOCX 29 kb)

## Appendix

### The GRM and IIF models

The probability of respondent *j* with latent ability level *θ*_*j*_ (the latent trait for respondent *j*) to choose response option *k* or higher (in our case, *k* = 0, 1, 2, 3, 4, 5) for item *i* is [45, 58]:

$$ \mathit{\Pr}\;\left({Y}_{ij}\ge k\left|{\theta}_j\right.\right)=\frac{\mathit{\exp}\left\{{\alpha}_i\left({\theta}_j\hbox{-} {b}_{ik}\right)\right\}}{1+\mathit{\exp}\left\{{\alpha}_i\left({\theta}_j-{b}_{ik}\right)\right\}}{\theta}_j\sim N\left(0,1\right) $$

where, *a*_*i*_ represents the discrimination of item *i*, and *b*_*ik*_ is the cut-point of boundaries between the *k*^*th*^ and *(k + 1)*^*th*^ options for item *i*, which can be considered as the difficulty of choosing option *k* or higher for item *i* [45, 58].

The information function *I*_*i*_*(θ)* for item *i* is:

$$ {I}_i\left(\theta \right)={\sum}_{k=1}^K{I}_{ik}\left(\theta \right){p}_{ik}\left(\theta \right) $$

where, *I*_*ik*_(*θ*) is the information function, for response option *k* of item *i*, which is defined as:

$$ {I}_{ik}\left(\theta \right)=-\frac{\partial^2\mathit{\log}{p}_{ik}\left(\theta \right)}{\partial {\theta}^2} $$

where, ∂ is the partial derivative symbol, and *p*_*ik*_(*θ*) is the probability of a respondent with the latent trait level *θ* choosing response option *k*, which depends on the GRM for item *i*.

## Abbreviations

CFA: Confirmatory factor analysis
CFI: Comparative fit index
CTT: Classical test theory
DCCT: Diabetes control and complications trial
DM: Diabetes mellitus
DQOL: Diabetes quality-of-life measure
EFA: Exploratory factor analysis
GRM: Graded response model
HRQoL: Health-related quality of life
IIF: Item information function
IRF: Item response function
IRT: Item response theory
RMSEA: Root mean square error of approximation
SD: Standard deviation
SRMR: Standardized root mean squared residual
T2DP: Type 2 diabetic patients
VAS: Visual analogue scale
