# Supplementary material for: Item reduction and validation of the Chinese version of diabetes quality-of-life measure (DQOL)
Source: Health Qual Life Outcomes. 2018 Apr 27;16:78. doi: 10.1186/s12955-018-0905-z (PMC5921810; doi:10.1186/s12955-018-0905-z)
Supplement: Supplementary file 1 — Original Chinese DQOL and short versions based on the CTT and IRT. (DOCX 29 kb) [file 12955_2018_905_MOESM1_ESM.docx]

**Additional file 1**

**Original Chinese DQOL and short versions based on the CTT and IRT**

| *Original Chinese DQOL* | | *Short version based on the CTT (32 items)* | *Short version based on the IRT (24 items)* |
| --- | --- | --- | --- |
| *请您对您以下各个方面(1-15)的主观满意程度进行评价：*  *（满意度：1.非常满意 2.满意 3.一般 4.不满意 5.非常不满意）*  *Satisfaction (1 Very satisfied, 2 satisfied, 3 General, 4 Dissatisfied, 5 Very dissatisfied)* | | | |
| 1 | 您对医生控制您的病情所花的时间满意吗？  How satisfied are you with the amount of time it takes to manage your diabetes? | √ | √ |
| 2 | 您对常规的体格检查所花的时间满意吗？  How satisfied are you with the amount of time you spend getting checkups? | √ | √ |
| 3 | 您对医生确定您的血糖水平所花的时间满意吗？  How satisfied are you with the time it takes to determine your sugar level? | √ | √ |
| 4 | 您对目前接受的治疗措施满意吗？  How satisfied are you with your current treatment? | √ | √ |
| 5 | 您对自己受限制的饮食满意吗？  How satisfied are you with the flexibility you have in your diet? | √ | 🞨 |
| 6 | 您对自己患糖尿病后给家庭带来的经济负担满意吗？  How satisfied are you with the burden your diabetes is placing on your family? | √ | √ |
| 7 | 您对自己关于糖尿病知识的了解程度满意吗？  How satisfied are you with your knowledge about your diabetes? | 🞨 | 🞨 |
| 8 | 您对自己的睡眠状况满意吗？  How satisfied are you with your sleep? | √ | 🞨 |
| 9 | 您对自己的社会关系和得到的友爱满意吗？  How satisfied are you with your social relationships and friendships? | √ | √ |
| 10 | 您对自己的性生活满意吗？  How satisfied are you with your sex life? | 🞨 | 🞨 |
| 11 | 您对自己的工作、学业和家庭生活满意吗？  How satisfied are you with your work, school, and household activities? | √ | √ |
| 12 | 您对自己的体型满意吗？  How satisfied are you with the appearance of your body? | √ | 🞨 |
| 13 | 您对自己每天能够用于锻炼身体的时间满意吗？  How satisfied are you with the time you spend on exercising? | √ | 🞨 |
| 14 | 您对自己的业余生活满意吗？  How satisfied are you with your leisure time? | √ | √ |
| 15 | 总的来说，您对自己的生活感到满意吗？  How satisfied are you with life in general? | √ | √ |
| *请您对糖尿病给您以下各方面(16-35)带来的影响进行评价：*  *（影响程度：1.从来没有 2.很少有 3.偶尔有 4.经常有 5.一直有）*  *Impact (1 Never, 2 Rarely, 3Occasional, 4 Often, 5 Always)* | | | |
| 16 | 您患糖尿病后对经常不得不接受治疗感到痛苦吗？  How often do you feel pain associated with the treatment for your diabetes? | √ | √ |
| 17 | 您经常对在公共场合下不得不谈及您的病情而感到尴尬吗？  How often are you embarrassed by having to deal with your diabetes in public? | √ | √ |
| 18 | 您经常有心慌、出虚汗、头昏、颤抖等低血糖反应吗？  How often do you have low blood sugar*(e.g. palpitation, sweating, dizziness, trembling)*? ^a^ | √ | 🞨 |
| 19 | 您经常感到身体不舒服吗？  How often do you feel physically ill? | √ | √ |
| 20 | 您经常觉得自己患糖尿病给您的家庭生活带来麻烦吗？  How often does your diabetes interfere with your family life? | √ | √ |
| 21 | 您经常晚上睡眠不好吗？  How often do you have a bad night's sleep? | √ | 🞨 |
| 22 | 您经常感到糖尿病限制了您的社会交往和友谊吗？  How often do you find your diabetes limiting your social relationships and friendships? | √ | √ |
| 23 | 您经常自我感觉良好吗？  How often do you feel good about yourself? | 🞨 | 🞨 |
| 24 | 您经常感到自己的饮食受到限制吗？  How often do you feel restricted by your diet? | √ | 🞨 |
| 25 | 您患糖尿病后性生活经常受到影响吗？  How often does your diabetes interfere with your sex life? | 🞨 | 🞨 |
| 26 | 您患糖尿病后经常被人劝阻不要骑车或从事打字员之类的工作吗？  How often does your diabetes keep you from *riding a bike* or being a typist? ^b^ | 🞨 | 🞨 |
| 27 | 您患糖尿病后身体锻炼经常受到影响吗？  How often does your diabetes interfere with your exercising? | √ | √ |
| 28 | 您患糖尿病后经常无力承担家庭义务吗？  How often do you miss household duties because of your diabetes? ^c^ | √ | √ |
| 29 | 您经常向别人解释糖尿病的危害吗？  How often do you find yourself explaining what it means to have diabetes? | 🞨 | 🞨 |
| 30 | 您患糖尿病后业余活动经常受到影响吗？  How often do you find that your diabetes interrupts your leisure-time activities? | √ | √ |
| 31 | 您患糖尿病后经常向别人诉说自己的病情吗？  How often do you tell others about your diabetes? | 🞨 | 🞨 |
| 32 | 您患糖尿病后经常被别人取笑吗？  How often are you teased because you have diabetes? | 🞨 | 🞨 |
| 33 | 您患糖尿病后经常感觉自己去洗手间的次数比别人多吗？  How often do you feel that because of your diabetes you go to the bathroom more than others? | √ | 🞨 |
| 34 | 经常发现自己隐瞒病情而去吃一些自己不应该吃的东西吗？  How often do you find that you eat something you shouldn't rather than tell someone that you have diabetes? | 🞨 | 🞨 |
| 35 | 您经常隐瞒自己一直有胰岛素副反应的事实吗？  How often do you hide from others the fact that you are having an insulin reaction? | 🞨 | 🞨 |
| *请您对以下方面(36-46)的忧虑程度进行评价：*  *（忧虑程度 I：1.从不担心 2.很少担心 3.偶尔担心 4.经常担心 5.总是担心）*  *Worry (1 Never, 2 Rarely, 3Occasional, 4 Often, 5 Always)* | |  |  |
| 36 | 您患糖尿病后经常为将来的婚姻状况感到忧虑吗？  How often do you worry about your *marriage*? ^d^ | 🞨 | √ |
| 37 | 您患糖尿病后经常为孩子的将来感到忧虑吗？  How often do you worry about your *children's future*? ^e^ | √ | √ |
| 38 | 您患糖尿病后经常为以后可能找不到理想的工作感到忧虑吗？  How often do you worry about whether you will not get a job you want? | 🞨 | 🞨 |
| 39 | 您患糖尿病后经常为以后可能得不到养老金或离退休金感到忧虑吗？  How often do you worry about whether you will be denied *pension*? ^f^ | √ | 🞨 |
| 40 | 您患糖尿病后经常为以后能否完成自己的继续教育感到忧虑吗？  How often do you worry about whether you will be able to complete your education? | 🞨 | 🞨 |
| 41 | 您患糖尿病后经常为将来可能会失业感到忧虑吗？  How often do you worry about whether you will lose your job? ^g^ | 🞨 | 🞨 |
| 42 | 您患糖尿病后经常为将来可能不能外出旅游感到忧虑吗？  How often do you worry about whether you will be able to take avacation or a trip? | √ | √ |
| 43 | 您患糖尿病后经常为将来可能会昏厥感到忧虑吗？  How often do you worry about whether you will pass out? | √ | √ |
| 44 | 您患糖尿病后经常为自己的体型与别人不同感到忧虑吗？  How often do you worry that your body looks different because you have diabetes? | √ | √ |
| 45 | 您患糖尿病后经常为自己可能会发生并发症感到忧虑吗？  How often do you worry that you will get complications from your diabetes? | √ | √ |
| 46 | 您患糖尿病后经常为有人不愿意和您一起外出感到忧虑吗？  How often do you worry about whether someone will not go out with you because you have diabetes? | √ | √ |
| English translation is provided after each Chinese item.  “√” indicates the item was kept; “🞨” indicates the item was removed from the scale.  a. The original English version was “How often do you have low blood sugar?”  b. The original English version was “How often does your diabetes keep you from driving a car or using a machine (e.g., a typewriter)?”  c. The original English version was “How often do you miss work, school, or household duties because of your diabetes?”  d. The original English version was “How often do you worry get married?”  e. The original English version was “How often do you worry have children?”  f. The original English version was “How often do you worry be denied insurance?”  g. The original English version was “How often do you worry about whether you will miss work?”  *DQOL* Diabetes quality-of-life, *CTT* Classical test theory, *IRT* Item response theory. | | | |
